# Maternal Environmental Contribution to Adult Sensitivity and Resistance to Obesity in Long Evans Rats

**DOI:** 10.1371/journal.pone.0013825

**Published:** 2010-11-03

**Authors:** Mariana Schroeder, Liat Shbiro, Timothy H. Moran, Aron Weller

**Affiliations:** 1 Psychology Department, Bar Ilan University, Ramat Gan, Israel; 2 Gonda (Goldschmied) Brain Research Center, Bar Ilan University, Ramat Gan, Israel; 3 Department of Psychiatry and Behavioral Sciences, Johns Hopkins University School of Medicine, Baltimore, Maryland, United States of America; AgroParisTech, France

## Abstract

**Background:**

The OLETF rat is an animal model of early onset hyperphagia induced obesity, presenting multiple pre-obese characteristics during the suckling period. In the present study, we used a cross-fostering strategy to assess whether interactions with obese dams in the postnatal environment contributed to the development of obesity.

**Methodology:**

On postnatal Day (PND)-1 OLETF and control LETO pups were cross-fostered to same or opposite strain dams. An independent ingestion test was performed on PND11 and a nursing test on PND18. Rats were sacrificed at weaning or on PND90, and plasma leptin, insulin, cholesterol, triglycerides and alanine aminotransferase (ALT) were assayed. Fat pads were collected and weighed and adipocyte size and number were estimated. Body weight and intake, as well as the estrous cycle of the female offspring were monitored.

**Principal Findings:**

During the suckling period, the pups' phenotype was almost completely determined by the strain of the mother. However, pups independently ingested food according to their genotype, regardless of their actual phenotype. At adulthood, cross fostered males of both strains and LETO females were affected in regard of their adiposity levels in the direction of the foster dam. On the other hand, OLETF females showed almost no alterations in adiposity but were affected by the strain of the dams in parameters related to the metabolic syndrome. Thus, OLETF females showed reduced liver adiposity and circulating levels of ALT, while LETO females presented a disrupted estrous cycle and increased cholesterol and triglycerides in the long term.

**Conclusions:**

The present study provides further support for the early postnatal environment playing a sex-divergent role in programming later life phenotype. In addition, it plays a more central role in determining the functioning of mechanisms involved in energy balance that may provide protection from or sensitivity to later life obesity and pathologies related to the metabolic syndrome.

## Introduction

Maternal nutritional and hormonal environment during pregnancy and lactation strongly influence the programming of life-long appetite and energy expenditure in the offspring [1]. In addition, the hormonal, neuronal and autocrine mechanisms that contribute to the maintenance of energy balance develop during this period, giving the perinatal environment high importance in determining the programming of later sensitivity or resistance to obesity [2].

Maternal obesity during pregnancy and lactation have been found to influence the long term phenotype of the offspring, predisposing them to develop increased adiposity, accompanied by higher leptin and glucose levels later in life [3],[4]. The postnatal environment can even modify a genetic tendency [5],[6]. For example, obesity-prone mice fostered to obesity-resistant dams displayed attenuated obesity, whereas obesity-resistant mice fostered to obesity-prone dams develop obesity and insulin resistance [7]. Similarly, although (diet-induced-) obesity-prone rat pups cross-fostered to obesity resistant dams remained obese, they had improved insulin sensitivity as adults; in contrast, obesity resistant rat pups cross-fostered to genetically obese dams showed a diet-induced increase in adiposity, reduced insulin sensitivity and changes in hypothalamic neuropeptide expression [8].

Otsuka Long Evans Tokushima Fatty (OLETF) rats are a model of non-insulin dependent diabetes mellitus (NIDDM) [9],[10] and early-onset hyperphagia- induced obesity [11],[12],[13],[14],[15]. They have a congenital defect in the expression of the cholecystokinin-1 (CCK_1_) receptor gene [16] leading to the lack of CCK_1_ receptors. In normal rats, CCK, a peripheral satiety signal, elicits the early appearance of the behavioral satiety sequence upon ingestion of dietary fat [17],[18]. OLETF rats lack this inhibitory control mechanism.

We have recently found that many pre-obese characteristics are evident in the OLETF strain soon after birth, throughout lactation and around adolescence. OLETF rats are born heavier [15], are hyperphagic during the lactation period as evidenced by increased intake in both independent ingestion and suckling tests [11],[13],[19] and display increased adiposity as a result of adipocyte hypertrophy [14]. Moreover, previous studies have demonstrated increased nursing in the OLETF strain, as a consequence of longer nursing bouts especially in the third postnatal week [13],[19]. This, together with an increased suckling capacity [13] and initiative on the side of the pups and maternal high fat milk on the side of the dams [19],[20], provides OLETF pups with an obesogenic perinatal environment (OLETF dams are obese and hyperphagic during pregnancy) that contributes to their life-long obese phenotype. In addition, we have noted sex differences in the profile of obesity development. Significant signs of obesity (sharp increase in white fat, adipocyte size and leptin) appear much earlier in the OLETF males (around PND48) than in the females (PND 90), suggesting different adiposity related turning-points between the sexes that can also be observed in the control (LETO) strain.

Thus, the early postnatal (and prenatal) environment appears to program subsequent obesity and provides a potential therapeutic target, which will hopefully translate into improved intervention strategies to affect the epidemic of obesity, a condition which, once manifest, has proven particularly hard to treat. In the present study, we aimed to examine this possibility by providing an obesogenic postnatal environment to genetically intact pups, and a normal postnatal environment to pups genetically predisposed to become obese. We have also focused on how these alterations in postnatal environment may differentially affect male and female offspring.

## Methods

### Subjects

OLETF and LETO rats were raised in the SPF facility of the Gonda Brain Research Center at Bar-Ilan University, Ramat-Gan, Israel. The original rats were received as a generous gift from the Tokushima Research Institute, Japan. OLETF and LETO offspring were housed together with their dams and litters until weaning and in pairs from then on. Polycarbonate cages (23.5 cm height ×26.5 cm width ×43 cm length) were used, with stainless steel wire lids and wood shavings as bedding material. Standard chow (2018S Teklad Global, 5% fat) and water were freely available. The animals were on a 12:12 hr light: dark cycle, with lights on at 06:00. Room temperature was maintained at 22+/−2°C. The pregnant females were checked daily for parturition. Newborn litters found until 12:00 hr each day were designated as born on that day (PND 0). On PND 1, litters were culled to 10 pups when a large litter was born. Litters with less than 8 pups were excluded from the experiment. Sex distribution was kept as equal as possible in each litter. At this point, entire (culled) litters were fostered to another dam, either from the same or the opposite strain. At weaning, all pups were weaned to standard chow. Six to seven litters were used per group.

The research protocol was approved by the Institutional Animal Care and Use Committee (permit number: 10-3-07), and it adhered to the guidelines of the American Psychological Association and the Society for Neuroscience.

**Figure 1:**
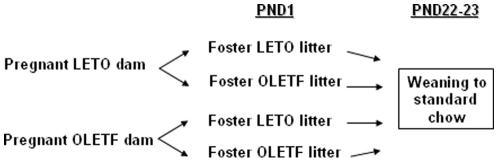
Experimental Design.

We labeled the groups as follows: The LETO in fostering group was named **LdLp** (LETO dam- LETO pups); the OLETF in fostering group was accordingly named **OdOp**, the LETO dam- OLETF pups group was named **LdOp** and the OLETF dam- LETO pups group was named **OdLp**. Please see figure 1 for the experimental design.

### Experimental procedure: Body weight (BW) and intake

Rats were weighed every fifth day from birth (PND1) until PND 90. Food intake was assessed daily starting at the time of weaning (PND 22).

### Independent ingestion test

Ten to twelve day old pups (animals coming from the same litter were put together) were held together in a cage containing bedding from the mothers' cage (to reduce stress) inside a humid incubator (33°C), for a deprivation period of 3 hours. Then, every pup was weighed, excretion was stimulated by a cotton swab, then the pup was reweighed and individually placed in a plastic beaker containing 2 ml of high-fat milk (UHT long life cream, 10% fat, Tnuva dairy, Israel) sweetened by the addition of sucrose to yield a 10% solution, warmed to 38°C and spread uniformly over tissue paper cut to fit the bottom of the beaker. The beaker with the pup inside it was put in a moist incubator maintained at 33°C. After 30 min, each pup was removed from the incubator, wiped dry with tissue paper, and weighed. Intake was measured by the change in BW that occurred during the 30-min test and is reported as percent BW gain. Six to eight pups were tested per litter.

### Nursing test

Pups were examined with their foster dam in their home cages, once, between PND 18–20. Experiments took place between 10:00 and 15:00 hr after a 4 hr separation from the dam. Pups were placed as a group in a cage containing bedding from the home cage and in a humid and warm incubator (33°C) for that period while the dams remained in their home cage. Excretion was induced with a cotton swab and then pups were weighed and individually identified with a permanent marker. After the isolation period, the pups were returned to the nest, scattered in the 4 corners of the cage (e.g., as in [13] and the following parameters were examined:

1. *Body weight* at the time of the test;
2. *Weight gain*: weight gain in percentage of BW after the nursing episode;
3. *Latency to start nursing*: time until the beginning of a nursing episode. The onset was determined as when the dam was crouching over her pups, with at least four of them under her ventrum and attached to her nipples for at least 2 min;
4. *Nursing episode length*.

### Tissue collection

Rats were sacrificed at weaning (PND22-3) or on PND90. On the day of sacrifice, rats were weighed and sacrificed between 11:00 AM and 2:00 PM. Interscapular brown adipose tissue (BAT), Retroperitoneal (Retro), Inguinal (IAT) and Epydidimal adipose tissues (EPY-only males) were collected from decapitated animals and weighed. A sample of IAT was cut and immediately frozen on dry ice for later analysis of adipocyte size and number. Samples were preserved at −80°C until analyzed. Liver adiposity was determined by using Double X-ray Absorption (DXA; Lunar Piximus II). Trunk blood for plasma analysis was collected in chilled heparinized vacutainer tubes coated with EDTA. Plasma was stored at −80°C until assayed.

### Leptin and insulin

Plasma leptin and insulin levels were assessed using commercial ELISA kits (R&D Systems and Linco, respectively) according to the manufacturers' instructions.

### Biochemical tests

Alanine aminotransferase (ALT) (an enzyme that is normally present in liver and heart cells, that is released into the blood when liver or heart are damaged), cholesterol and triglycerides were assessed from plasma by a chemistry analyzer (Hitachi 917, Roche diagnostics, Herzliya medical center). Around PND80 rats were anesthetized after overnight fasting with pentobarbital (200 mg/ml) and Assival (5 mg/ml) (1∶1). Blood was obtained from the tail and glucose levels were determined with a glucometer (Accu-check active, Roche, Germany).

### Histology

Samples of the inguinal white adipose tissue (IAT) were used to characterize adipocyte cell size and number. Tissues were sectioned to 8 micrometers by a Cryostat (Leyca) at −35°C and mounted on glass slides. Digital photographs were rapidly taken using the ACT1 program, at ×200 magnification. For each inguinal fat pad examined, 10–20 pictures were taken from 3 different zones of the sample, at least 100 micrometers distance from each other. Adipocyte size parameters were derived from 4 to 8 representative cells from each picture, depending on the size of the cell, using the public domain National Institutes of Health Scion image program. For each animal, at least 80 cells were analyzed. Representative cells chosen presented a smooth and clear membrane, with no surrounding granulation. A similar methodological approach has been described elsewhere [14],[21]. The estimated number of cells per fat pad was calculated using the average diameter, a density conversion factor (0.915 g/cc), and the mass of the fat pads, as previously described [22],[23]. The number of cells was normalized to rat BW for data analysis. Cell analysis was performed by 2 investigators blind to the experimental groups.

### Estrous cycle

The estrous cycle stage of 50–80 day old females was examined daily in the morning. Males were handled in parallel to provide similar experimental conditions to both sexes. Vaginal cytology samples were collected by introduction and immediate extraction of a small amount of phosphate buffer with a micropipette in the rat's vagina. The stage of the estrous cycle (diestrous 1 or metaestrous, diestrous 2, pro-estrous or estrous) was determined by examining the appearance and abundance of cells within the vaginal cytology samples. Metaestrous was characterized by leukocytes and clusters of cornified cells, diestrous 2 was characterized by leukocytes and nucleated epithelial cells, pro-estrous was characterized primarily by nucleated epithelial cells, and estrous was characterized by an abundance of cornified cells. In 5-day cycles, either Diestrous 2 or Estrous appeared twice. Six or seven cycles were analyzed per female and food intake was also assessed across the different stages of the estrous cycle.

## Results

### Statistical approach

Group differences in BW and intake were analyzed by repeated measures ANOVA comparing the 2 groups within each pup-genotype (LETO/OLETF) and sex. Group differences at particular days of measurement were followed-up by one-way ANOVA. Overall, we focused on within-sex fostering influences (e.g., male LdLp vs. male OdLp) given that sex differences and strain differences are large and already known [23]. For the independent ingestion and the nursing test, MANOVA for each pup-strain was performed. For presentation only, we included same-strain males and females in the same figures. All data of pups from the same litter were averaged and considered n = 1, with the exception of the estrous cycle data, where 2 females per litter were included in the analysis.

### BW and Intake

**Figure 2:**
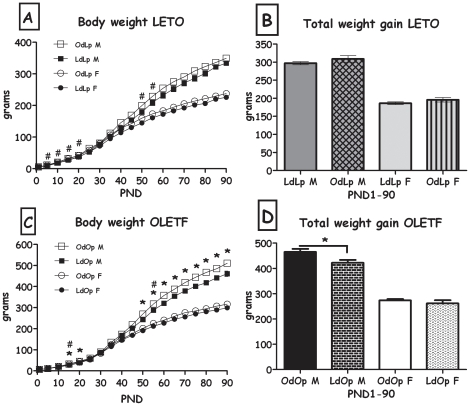
Body weight. **A**: LdLp and OdLp rats' body weight in grams from PND 22 until 90 and **B**: total body weight gain in grams from birth until 90 (both sexes). *p<0.05 for the males, #p<0.05 for the females (OdLp significantly different than LdLp). **C**: OdOp and LdOp rats' body weight in grams from PND 22 until 90 and **D**: OdOp and LdOp rats' total body weight gain in grams from birth until 90 (both sexes). *p<0.05 for the males, #p<0.05 for the females (LdOp significantly different than OdOp). Data are presented in means and SEM. N = 6–7 litters per group.

The body weights of female cross-fostered OdLp and LdLp differed during lactation, where OdLp females weighed significantly more than controls. After weaning, OdLp females recovered BW and total weight gain during the post-weaning period did not differ from controls (age x fostering group interaction (F(18,198) = 2.81, p<0.001, Figures 2a&b). The weight of male LETO pups was not affected by fostering condition despite a significant age x fostering group interaction (F(18,198) = 2.44, p<0.001). Overall, cross-fostering did not affect the body weights of female OLETF pups, from fostering to adulthood. In contrast, male LdOp pups showed reduced body weight compared to OdOp controls (F(1,11) = 7.12, p<0.05 for the group main effect and F(18,198) = 6.33, p<0.001 for the group X time interaction) and reduced weight gain during the post-weaning period (differences based on one-way ANOVA, Figures 2c&d). Specific ages in which this difference was significant are shown in Figure 2C.

**Figure 3:**
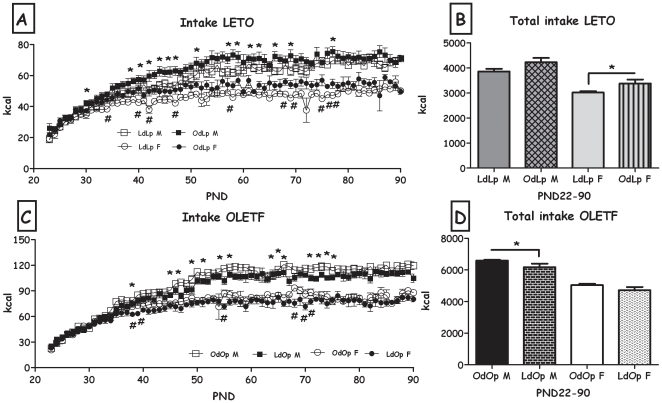
Voluntary intake. **A**: LdLp and OdLp rats' daily intake in kcal from PND 22 until 90 and **B**: total intake (both sexes). Data are presented in means and SEM. *p<0.05 for the males, #p<0.05 for the females (OdLp significantly different than LdLp). **C**: OdOp and LdOp rats' daily intake in kcal from PND 22 until 90 and **D**: total intake (both sexes). Data are presented in means and SEM. *p<0.05 for the males, #p<0.05 for the females (LdOp significantly different than OdOp). N = 6–7 per group.

Regarding food intake, OdLp males showed a different intake trajectory than controls (age x group interaction, F(67,670) = 1.46, p<0.05), but no overall effect of adoption on overall intake (Figures 3a&b). OdLp showed significantly higher intake levels than LdLp controls (F(1,10) = 5.13, p<0.05 for the adoption (group) effect and F(67,670) = 1.37, p<0.05 for the age x group interaction, Figures 3a&b). In the OLETF strain, females were not affected by adoption, but LdOp males consumed significantly less food than OdOp controls (F(1,11) = 4.99, p<0.05 for the adoption effect and F(67,737) = 1.65, p<0.001 for the age x group interaction, Figures 3c&d). When normalized to body weight, intake did not differ between the groups (data not shown).

### Independent ingestion test

**Figure 4:**
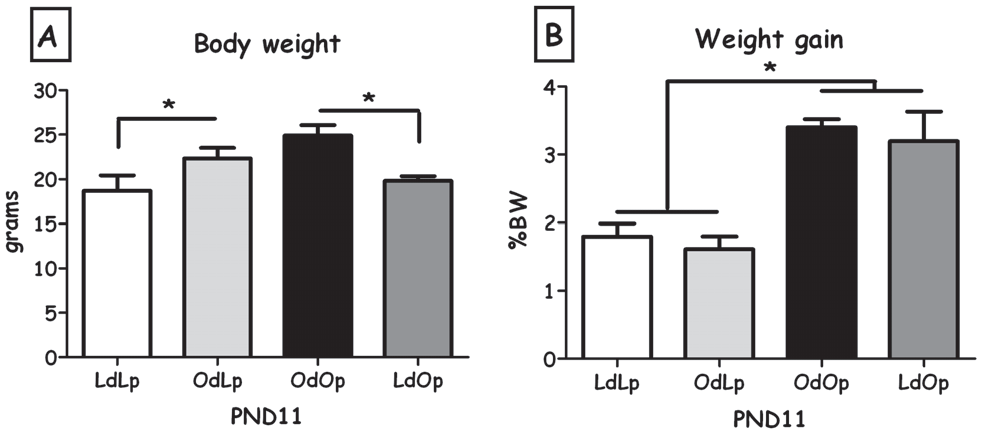
Independent ingestion test. A: Pups' body weight in grams at the time of the test (PND10-12). **B**: Pups' body weight gain (percentage of body weight) after the 30-min independent ingestion test. Data are presented in means and SEM. *p<0.05 for pup-strain comparison between cross and in-fostering groups. N = 6–7 per group.

At the time of the test (PND11), OdLp pups tended to weigh more than LdLp pups and LdOp pups weighed significantly less than OdOp pups (F(1,12) = 15.90, p<0.01, Figure 4a). In contrast, weight gain in the test (normalized to BW) was a function of genotype as can be appreciated by the higher amounts of food consumed by OLETF pups, compared to LETO pups of both fostering conditions (Figure 4b). Latency to start feeding did not differ between the groups (not shown). Data of males and females were combined, since no statistical differences were found between the sexes, as in previous studies using this test at this age.

### Nursing test

**Figure 5:**
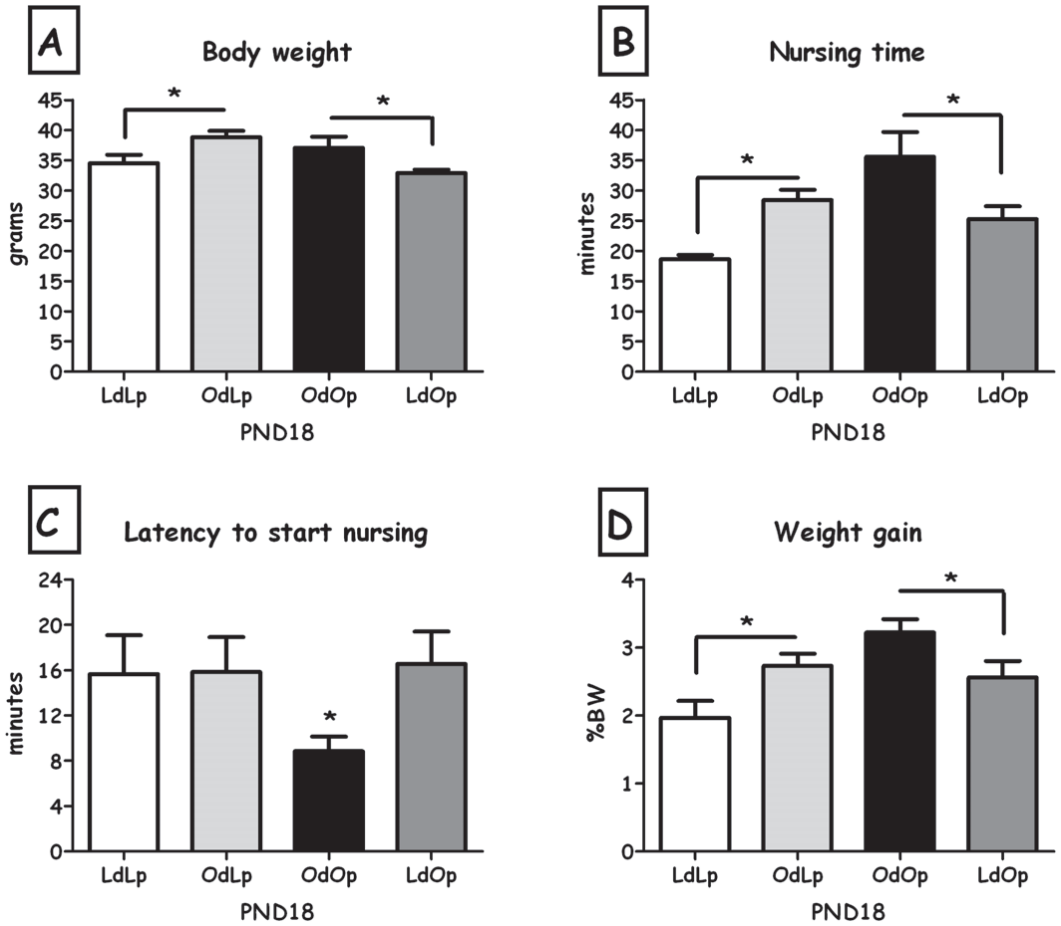
Nursing test. **A**: Pups' body weight in grams at the time of the test (PND18); **B**: Nursing episode duration in minutes; **C**: Latency to start nursing in minutes; **D**: Pups' body weight gain (percentage of body weight) after nursing test (D). Data are presented in means and SEM. *p<0.05 for pup-strain comparison between cross and in-fostering groups. N = 6–7 per group.

On PND18, OdLp pups weighed significantly more than LdLp pups and LdOp pups weighed significantly less than OdOp pups (both F>4.92, p<0.05) (Figure 5a). Weight gain (normalized to BW) from nursing was significantly higher in OdLp pups compared to LdLp pups, while LdOp pups showed decreased intake compared to OdOp pups (both F>5.03, p<0.05, Figure 5d). Latency to start nursing was shorter in the OdOp group compared to LdOp (F(1,12) = 6.12, p<0.05, Figure 5c). Nursing time was longer in the OdLp group compared to LdLp pups and shorter in the LdOp group compared to OdOp pups (both F>5.03, p<0.05, Figure 5c). Data from males and females were combined, since no statistical differences were found between the sexes.

### Adiposity measures

#### Weaning age

**Figure 6:**
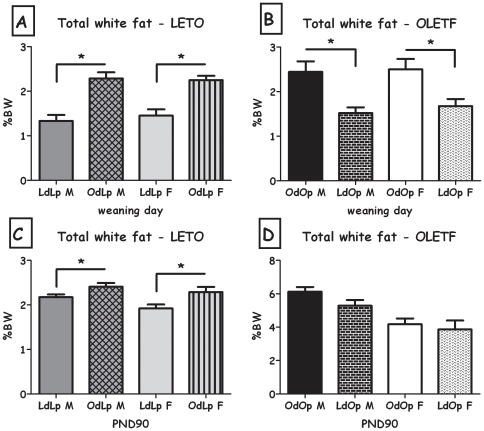
Total white fat (expressed as percent of BW). **A**: LdLp pups vs. OdLp pups at weaning; **B**: OdOp pups vs. LdOp pups at weaning; **C**: LdLp pups vs. OdLp pups on PND90; **D**: OdOp pups vs. LdOp pups on PND90. Data are presented in means and SEM. *p<0.05 for pup-strain comparison between same-sex cross and in-fostering groups. N = 6–7 per group.

**Figure 7:**
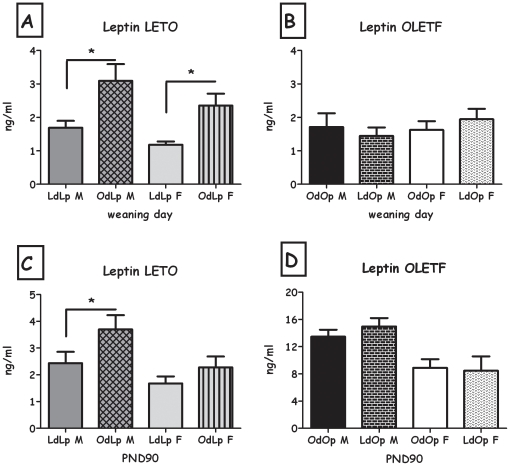
Plasma leptin levels. **A**: LdLp pups vs. OdLp pups at weaning; **B**: OdOp pups vs. LdOp pups at weaning; **C**: LdLp pups vs. OdLp pups on PND90; **D**: OdOp pups vs. LdOp pups on PND90. Data are presented in means and SEM. *p<0.05 for pup-strain comparison between same-sex cross and in-fostering groups. N = 5–6 per group.

**Figure 8:**
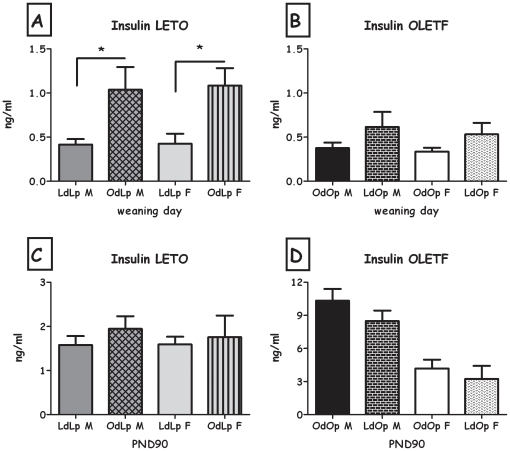
Plasma insulin levels. **A**: LdLp pups vs. OdLp pups at weaning; **B**: OdOp pups vs. LdOp pups at weaning; **C**: LdLp pups vs. OdLp pups on PND90; **D**: OdOp pups vs. LdOp pups on PND90. Data are presented in means and SEM. *p<0.05 for pup-strain comparison between same-sex cross and in-fostering groups. N = 5–6 per group.

Overall, body fat, leptin and insulin levels in weaning-age pups were affected by the foster dam's genotype. This pattern was observed for the total weight of the white fat pads (expressed as percent of the rat's BW), and separately for retroperitoneal and inguinal fat pads, both in males and in females and epydidimal fat in OLETF males (all F>5.06; all p<0.05) (Tables 1&2). In addition, OdLp rats displayed greater amount of total white fat pads, compared to LdLp (Figure 6a). Similarly, cross-fostering LETO pups to an OLETF dam (OdLp) resulted in significantly higher levels of leptin (Figure 7a) and insulin (Figure 8a), in both males and females (all F>6.63; all p<0.05). Leptin and insulin levels did not differ between same-sex LdOp and OdOp groups (Figures 7b&8b). Similarly, liver adiposity was significantly greater in OdLp females compared to LdLp controls (p<0.05), an effect not found in the males or in the OLETF strain weanlings (Table 3). In contrast to the white fat pads, where the pattern of adiposity reflected the dam's genotype, BAT levels (expressed as percent of the rat's BW) at weaning were determined by the strain of the pups and were not affected by adoption (Tables 1&2).

**Table 1:** Weight of the different fat pads in the LETO strain.

WEANING DAY	LDLP M		ODLPM		LDLP F		ODLP F	
	grams	%BW	grams	%BW	grams	%BW	grams	%BW
Retroperitoneal	0.15±0.03	0.31±0.05	**0.39±0.05** *	**0.73±0.07#**	0.16±0.03	0.35±0.03	0.35±0.03*	**0.65±0.04#**
Inguinal	0.47±0.06	1.02±0.09	**0.82±0.08** *	**1.56±0.07#**	0.50±0.06	1.11±0.09	0.87±0.10*	**1.60±0.07#**
Epididymal	0.05±0.01	0.10±0.02	0.09±0.01	0.15±0.02	________	________	________	________
Brown	0.29±0.01	0.63±0.03	**0.39±0.04** *	0.71±0.02	0.28±0.01	0.63±0.01	0.34±0.03	0.64±0.01

PND90	LDLP M		ODLPM		LDLP F		ODLP F	
	grams	%BW	grams	%BW	grams	%BW	grams	%BW
Retroperitoneal	1.46±0.06	0.44±0.02	**1.90±0.14** *	**0.54±0.02#**	1.16±0.08	0.51±0.03	**1.65±0.17** *	**0.69±0.05#**
Inguinal	5.83±0.20	1.74±0.05	6.51±0.38	1.86±0.07	3.25±0.17	1.41±0.06	3.81±0.25	1.60±0.06
Epididymal	1.98±0.10	0.59±0.03	2.25±0.20	0.64±0.04	________	________	________	________
Brown	0.48±0.02	0.14±0.01	0.48±0.01	0.14±0.00	0.35±0.02	0.15±0.00	0.39±0.02	0.17±0.01

Data are presented in mean and SEM.

*p<0.05, for significant group differences within each strain and sex (in grams);

#p<0.05, for significant group differences within each strain and sex (in percentages).

**Table 2:** Weight of the different fat pads in the OLETF strain.

WEANING DAY	ODOP M		LDOP M		ODOP F		LDOP F	
	grams	%BW	grams	%BW	grams	%BW	grams	%BW
Retroperitoneal	0.36±0.04	0.65±0.06	**0.15±0.03** *	**0.32±0.06#**	0.34±0.05	0.64±0.08	**0.18±0.03** *	**0.39±0.08#**
Inguinal	1.01±0.13	1.80±0.18	**0.57±0.04** *	**1.20±0.07#**	0.98±0.11	1.86±0.16	**0.59±0.05** *	**1.28±0.09#**
Epididymal	0.07±0.01	0.12±0.01	**0.04±0.00** *	**0.08±0.01#**	________	________	________	________
Brown	0.24±0.04	0.43±0.06	0.22±0.02	0.47±0.04	0.19±0.01	0.35±0.02	0.20±0.01	**0.43±0.02#**

PND90	ODOP M		LDOP M		ODOP F		LDOP F	
	grams	%BW	grams	%BW	grams	%BW	grams	%BW
Retroperitoneal	8.35±0.48	1.60±0.07	**6.31±0.66** *	1.34±0.11	4.87±0.34	1.46±0.08	3.53±0.65	1.12±0.16
Inguinal	23.55±1.41	4.52±0.21	**18.37±1.50** *	3.97±0.23	10.57±0.71	3.17±0.18	8.42±1.49	2.70±0.38
Epididymal	8.48±0.52	1.63±0.07	**6.37±0.36** *	**1.36±0.07#**	________	________	________	________
Brown	1.03±0.09	0.20±0.01	0.86±0.07	0.20±0.00	0.76±0.06	0.23±0.02	0.70±0.10	0.23±0.02

Data are presented in mean and SEM.

*p<0.05, for significant group differences within each strain and sex (in grams);

#p<0.05, for significant group differences within each strain and sex (in percentages).

**Table 3:** Biochemical measurements in plasma and liver adiposity.

WEANING DAY	LETO STRAIN				OLETF STRAIN			
	LDLP M	ODLP M	LDLP F	ODLP F	LDLP M	ODLP M	LDLP F	ODLP F
Cholesterol (mg/dl)	119.0±15.69	146.4±12.55	103.4±12.11	**140.0±3.33** *	111.0±3.94	109.6±4.98	114.6±5.33	103.2±6.34
Triglycerides (mg/dl)	130.4±27.09	181.4±32.34	110.4±23.49	11.6±4.32	72.8±9.10	73.2±2.85	88.8±8.35	66.4±8.90
ALT (IU/L)	93.2±14.4	95.2±8.36	75.4±12.48	76.0±3.35	82.49±3.08	74.8±3.21	98.0±5.50	**72.6±11.00** *
Liver adiposity (%)	10.77±1.01	9.23±0.45	7.70±0.48	**10.37±0.53** *	9.87±1.23	7.87±0.56	8.39±0.94	8.92±0.69

PND90	LETO STRAIN				OLETF STRAIN			
	LDLP M	ODLP M	LDLP F	ODLP F	LDLP M	ODLP M	LDLP F	ODLP F
Cholesterol (mg/dl)	109.0±5.31	114.2±5.03	93.0±5.76	**12.75±6.98** *	133.6±11.99	153.4±3.31	108.8±9.57	113.4±5.18
Triglycerides (mg/dl)	96.6±6.83	114.6±8.93	78.6±12.44	**95.0±23.50** *	338.2±34.85	533.4±90.55	406.0±106.47	287.4±54.68
ALT (IU/L)	4.67±0.61	69.6±3.20	52.8±5.64	57.25±7.76	68.0±8.41	**94.2±12.03** *	96.6±6.44	**65.2±5.85** *
Liver adiposity (%)	136.65±4.14	6.35±1.41	5.86±1.04	5.29±0.48	8.89±0.75	**6.38±0.64** *	7.47±0.56	**3.92±0.80** *

Data are presented in mean and SEM.

*p<0.05, for significant group differences within each strain and sex.

**Figure 9:**
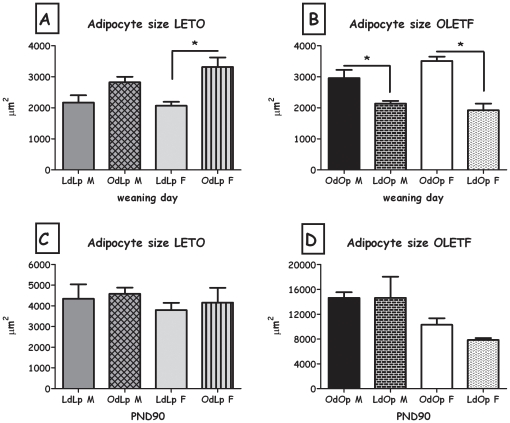
Adipocyte size calculated from inguinal adipose tissue. **A**: LdLp pups vs. OdLp pups at weaning; **B**: OdOp pups vs. LdOp pups at weaning; **C**: LdLp pups vs. OdLp pups on PND90; **D**: OdOp pups vs. LdOp pups on PND90. Data are presented in means and SEM. *p<0.05 for pup-strain comparison between same-sex cross and in-fostering groups. N = 4–6 per group.

**Figure 10:**
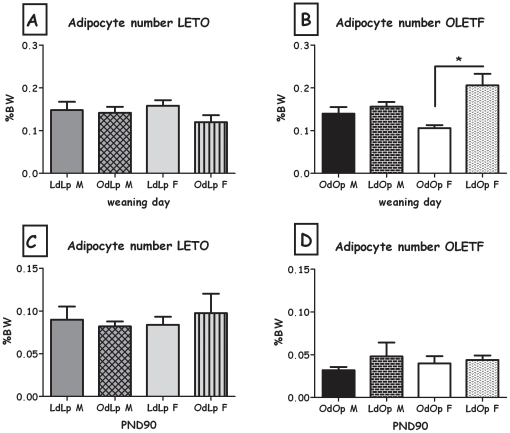
Estimation of the adipocyte number (expressed as percent of BW). **A**: LdLp pups vs. OdLp pups at weaning; **B**: OdOp pups vs. LdOp pups at weaning; **C**: LdLp pups vs. OdLp pups on PND90; **D**: OdOp pups vs. LdOp pups on PND90. Data are presented in means and SEM. *p<0.05 for pup-strain comparison between same-sex cross and in-fostering groups. N = 4–6 per group.

The overall pattern was also evident in adipocyte cell size, both in males and in females (Figures 9a&b). Besides the OdLp males, all changes in cell size were significant (all F>8.74; all p<0.05). Cell number was significantly greater in female LdOp weanlings compared to OdOp (F(1,8) = 13.12, p<0.01), there were no significant differences in the males or the LETO strain (Figures 10a & b).

#### Adulthood

At PND90, the genotype of the pups seemed to be the major determinant of adiposity, with OdLp similar to LdLp pups and LdOp similar to OdOp pups. However, OdLp males and females showed larger retroperitoneal fat pads and total white fat (expressed as percent of the rat's BW) compared to LdLp controls (both F>5.0; both p<0.05; Table 1& Figure 6c). In the OLETF strain, LdOp males showed reduced epydidimal (F(1,11) = 7.25, p<0.05; Table 2).

LdOp males and females had reduced liver adiposity compared to OdOp (both F>6.47; both p<0.05; Table 3). Long term effects on leptin levels were only evident in OdLp males, which presented higher levels than controls (F(1,18) = 9.15, p<0.01, Figure 7c). No effects were found in plasma insulin, adipocyte size and number in any of the groups (Figures 8c&d, 9c&d and 10c&d).

### Biochemical tests in plasma

At weaning, no significant effects were found among the groups in the OLETF strain in any of the parameters examined (Table 3). Results were similar in the LETO strain with the exception of OdLp females, which had significantly higher cholesterol levels than LdLp controls (F(1,8) = 8.50, p<0.05); Table 3).

On PND90, a significant effect of cross fostering on triglyceride and cholesterol levels was found in the OdLp females (both F>4.90, p<0.05; Table 3) compared to LdLp controls. LETO and OLETF males showed no long term effects on these parameters. LdOp females presented lower ALT levels (F(1,8) = 13.03, p<0.01) and tended to lower cholesterol levels compared to OdOp females (Table 3). No significant effects were found among the groups in glucose levels (Table 3).

### Estrous cycle

**Figure 11:**
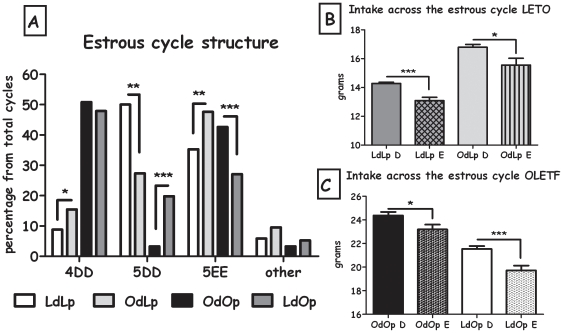
Estrous cycle parameters. **A**: Females' estrous cycle structure. Data are presented in percentages of total cycles examined. **p<0.01, ***p<0.001 for pup-strain comparison between cross and in-fostering groups. 4D: 4 day cycles, 5DD: 5day double estrous cycle and 5EE: 5 day double estrous cycles. **B**: Intake (kcal) across the cycle in the LETO strain and **C**: Intake (kcal) across the cycle in the OLETF strain. Data are presented mean and SEM.

As shown in Figure 11, cross fostering significantly affected the structure of the estrous cycle. In OdLp rats compared to LdLp rats (chi-square = 9.75, df = 2, p<0.01) the amount of 4-day cycles was increased, 5-day double diestrous cycles were less frequent and 5-day double estrous cycles were increased. In the OLETF strain, changes in the estrous cycle were also evident (chi-square = 17.70, df = 2, p<0.001). Cross fostering did not change the frequency of 4-day cycles in LdOp compared to OdOp females, but significantly increased their frequency of 5-day double estrous while reducing the occurrence of 5-day double estrous cycles. Overall, the fostering manipulation changed the estrous cycle structure according to the strain of the dam (Figure 11a). Analysis of the intake across the cycle was performed on data from the diestrous and estrous phases. Pro-estrous was excluded due to insufficient data. Overall caloric intake differed among the groups both in the diestrous (F(3,472) = 511.42, p<0.001) and the estrous phases (F(3,178) = 158.76, p<0.001). Specifically, OdLp females consumed more food than LdLp in both phases, while LdOp females consumed less food than OdOp in both phases (Duncan's test, p<0.05) (Figures 11b&c). Intake decrease from D to E (in percentages) was calculated between the groups, showing that LdOp had a sharper intake decrease than OdOp females in the estrous phase (8.43% vs. 4.87%) and OdLp presented a more moderate decrease than LdLp females (7.33% vs. 8.30%).

## Discussion

The present study was designed to evaluate the short and long term influence of the postnatal environment on the food intake, body weight and adiposity of normal and genetically hyperphagic rats. We have recently characterized the early developmental stages of obesity in the OLETF strain: Pups (both males and females) display many pre-obese characteristics such as increased body weight, intake and adiposity [13],[14],[15], soon after birth. OLETF pups also exhibited an avid appetite, reflected by their increased suckling efficiency in the first PN week as well as explicit initiative in inducing nursing bouts in the third PN week [13],[19]. In addition, an examination on the maternal (nursing) behavior in this strain also revealed a significant contribution from the side of the dam to the pre-obese phenotype of the pups. OLETF dams, when rearing their own pups, surrender to the pups' nursing demands resulting in longer nursing bouts (when rearing lean pups this nursing frequency declines), which together with their high fat milk results in overweight pups [13],[19].

Results from the current study, add further support to the view that the early pre-obese phenotype of the OLETF pups requires the interaction between the obese dam and the pup. While the lean foster dams' capacity to override the pups' genotype was undeniable during the suckling period, results of the nursing test (where weight gain of both fostering groups represented the average between regular LETO and OLETF pups) and especially of the independent ingestion test revealed that when given the chance, pups will consume food according to their own genotype, regardless of their present phenotype. Thus, following weaning from the foster dam, the pups gradually returned to their genetically predisposed adiposity profile.

Over the long term, the effects of the cross fostering were far less evident. Overall, genetically obese rats previously raised by lean mothers became obese. However, there were some lasting effects. LdOp males reached the end of the study weighing significantly less than OdOp males, and this was a result of a decrease in voluntary food intake. While still obese, LdOp males had less epididymal fat (suggesting reduced visceral fat) and reduced liver adiposity. Since large amounts of visceral fat are among the main causes of the metabolic syndrome and insulin resistance [24],[25], the results from the cross-fostered OLETF males suggest that modification of the early environment can have positive long term health consequences.

In contrast to the males, any short term effects of cross fostering on adiposity essentially disappeared by 90 days of age in female LdOp rats. However, there were lasting changes of cross fostering on the estrous cycle structure and decreased food intake in the estrous phase of the cycle together with a reduction in liver fat and circulating ALT levels, implying effects on aspects of the metabolic syndrome rather than on simple obesity and adiposity. The estrous cycle structure is usually abnormal in the OLETF females [26],[27], as is their intake across the cycle [26] and they also have fertility problems that are apparently linked to high levels of circulating leptin [27]. In OLETF rats, post-weaning leptin levels are high [10],[26], and in the pre-weaning period a significant leptin surge has been observed [14]. While this early surge of leptin has been related to later life leptin resistance [28], it has never been linked to fertility problems or changes in the estrous cycle structure but may be of relevance to the determination of their cycle and later fertility. This appears to be especially true given that OdLp females presented an estrous cycle similar in structure to regular OLETF females and over consumed food both in the diestrous and estrous phases of their cycle (maybe resulting in high cholesterol, triglycerides and white fat levels despite being lean and with low leptin levels). We will examine this possibility in future studies.

The contribution of an obesogenic postnatal environment to later body composition changes becomes clear when examining the results of the adoption on the LETO strain. OdLp males presented high adiposity as adults and had a significant (sustained) increase in circulating leptin levels. The relatively high levels of leptin and adiposity caused by the foster OLETF dam may have induced some level of leptin resistance, which can result from early life high levels of leptin [28],[29],[30]. This is especially true for the OdLp females, which presented (relative) hyperphagia during the post-weaning period. OdLp males and females showed a significant increase in retroperitoneal (and overall white) fat tissue. High levels of fat intake during lactation have previously been linked to increased retroperitoneal fat pad weight in the offspring [31].

In summary, the present study provides further support for the early postnatal environment playing a sex-divergent role in programming later life food intake, body weight, and adiposity. In addition, it plays a more central role in determining the functioning of mechanisms involved in energy balance that may provide some protection from or sensitivity to later life obesity and pathologies related to the metabolic syndrome. The perinatal environment provides a potential therapeutic target, and focusing on this specific developmental stage may translate into improved interventional strategies to stem the growing epidemic of obesity.
